# Bridging the Knowledge-Practice Gap in Breast Cancer Screening Among Non-medical Female University Students in Riyadh: A Cross-Sectional Study

**DOI:** 10.7759/cureus.115991

**Published:** 2026-09-09

**Authors:** Mazin Abdelgadir, Amaal Almohaimeed, Elaf Abalkhail, Haroon Wasson, Maria Alrafi, Lujain Bamehriz, Talal Alenazi, Enas Bedaiwi, Arwa Alanazi, Rahaf Aljejakli, Amen Bawazir, Mohammed Alghamdi

**Affiliations:** 1 College of Medicine, AlMaarefa University, Riyadh, SAU; 2 College of Medicine, Princess Nourah bint Abdulrahman University, Riyadh, SAU; 3 Department of Obstetrics and Gynecology, King Fahad Medical City, Riyadh, SAU; 4 College of Medicine, King Saud University, Riyadh, SAU; 5 College of Medicine, Alfaisal University, Riyadh, SAU; 6 Community Medicine Unit, Department of Basic Medical Sciences, College of Medicine, AlMaarefa University, Riyadh, SAU; 7 Department of Oncology Services, AlHammadi Hospitals Group, Saudi Society of Gastrointestinal Oncology, Riyadh, SAU

**Keywords:** breast cancer, non-medical university students, risk factors, saudi arabia, screening awareness, screening practices

## Abstract

Background

Breast cancer is the most prevalent female malignancy in Saudi Arabia and globally, with early detection being the cornerstone of improved survival. This study assesses breast cancer screening awareness, knowledge of risk factors and signs, and screening practices among non-medical female university students in Riyadh, Saudi Arabia.

Methods

A cross-sectional study (July-September 2025) enrolled 679 non-medical female students aged 18 years and above from four Riyadh universities using multistage sampling. Data were collected using a structured, bilingual 58-item questionnaire adapted from previous studies, culturally modified, pilot-tested, and reviewed by experts. Knowledge and practice domains were classified as sufficient when scores exceeded 60% and insufficient when scores were ≤60%. Knowledge scores were analyzed using Mann-Whitney U and Kruskal-Wallis tests with Bonferroni correction; chi-square tests and multivariate logistic regression identified significant predictors.

Results

Despite 85% (n = 578) demonstrating sufficient attitudes toward screening, only 44% (n = 299) had adequate general awareness, 19% (n = 132) had sufficient knowledge of risk factors and signs, and 23% (n = 158) achieved sufficient screening practice. The median knowledge score was 19/43 (IQR: 16-22). Family history (65.5%, n = 445) and increasing age (56.7%, n = 385) were the most recognized risk factors, while early puberty (16.2%, n = 110) and nulliparity (23.6%, n = 160) were the least. While 90.9% (n = 617) identified a breast mass as a sign, only 5.9% (n = 40) correctly recognized that a painful mass is not a typical early presentation. Breast self-examination and clinical breast examination uptake were low at 40.4% (n = 274) and 23.4% (n = 159), respectively. However, these screening practice findings should be interpreted considering the young age distribution of participants, with many being below the recommended age for routine mammography screening. AlMaarefa University affiliation was the strongest predictor of knowledge (OR = 2.718; 95% CI: 1.398-5.282; p = 0.003) and screening practice (OR = 5.488; 95% CI: 2.863-10.519; p < 0.001), with family history also predicting sufficient screening practice (OR = 2.384; 95% CI: 1.488-3.819; p < 0.001).

Conclusion

A striking knowledge-attitude-practice gap exists in this population. Universities are underutilized platforms for breast cancer health education, and structured, culturally sensitive interventions are urgently needed to convert attitudinal willingness into consistent screening practice.

## Introduction

Breast cancer represents the most commonly diagnosed malignancy among women globally and ranks as a leading cause of cancer-related mortality worldwide [1]. In 2020 alone, over 2.26 million incident cases were reported, placing an immense burden on healthcare systems and underscoring the urgency of preventive and early-detection strategies [2]. Projections from the American Cancer Society indicate that, absent significant changes in incidence trends, the global burden of breast cancer may approach 3.2 million new cases annually by 2050, a trajectory that demands immediate, coordinated public health action [3]. This projected increase highlights the importance of strengthening evidence-based prevention, early detection, and diagnostic strategies, particularly in developing countries [3]. In lower- and middle-income countries, as well as in rapidly developing economies, this urgency is amplified by the compounding effects of late presentation, limited health literacy, and insufficient screening infrastructure [4].

In the Kingdom of Saudi Arabia (KSA), breast cancer is the most prevalent malignancy among women, mirroring global epidemiological trends [5]. According to the Cancer Incidence Report of the Saudi Cancer Registry 2023, 56.8% of cases were diagnosed at the localized stage, 28.8% at regional involvement, and 13.6% with distant metastases, a staging distribution that reflects ongoing challenges in early detection and timely clinical engagement [5]. Breast cancer early detection strategies include population-based screening approaches, clinical evaluation of breast symptoms, and breast health awareness practices. While mammography remains the primary population-based screening modality supported by current evidence, breast self-examination (BSE) and clinical breast examination (CBE) represent additional approaches related to breast awareness and clinical assessment. Uptake of breast cancer early detection practices remains suboptimal across multiple population groups in KSA [6-9]. It is important to distinguish between breast awareness, diagnostic evaluation of breast symptoms, and population-based screening. Breast awareness refers to an individual's familiarity with the normal appearance and feel of their breasts and recognition of potential abnormalities that warrant medical evaluation. Diagnostic evaluation involves clinical assessment of individuals presenting with breast symptoms or abnormalities. In contrast, population-based screening refers to the systematic evaluation of asymptomatic individuals within an eligible age group, with mammography currently representing the primary evidence-supported screening modality for reducing breast cancer mortality [6-9].

The knowledge-practice gap, the divergence between what individuals know about a health issue and what they do in response, is particularly pronounced in the context of breast cancer screening in Saudi Arabia. A study assessing breast cancer awareness, knowledge, and engagement with early detection practices, including BSE and mammography, among 385 female residents in Arar City found that, despite generally high awareness levels, only one-third performed BSE and one-fifth had ever undergone mammography [10]. Similar patterns have been described in Jeddah and the Al Baha region, suggesting a nationally prevalent phenomenon rather than a localized anomaly [3-9]. The predominant barriers identified across these studies include lack of perceived necessity, misconceptions about screening frequency and method, fear of cancer discovery, social stigma, and logistical difficulties accessing screening services.

Non-medical university students occupy a unique and underexplored position in this public health landscape [11]. Unlike their peers enrolled in healthcare-related programs, non-medical students lack structured, curriculum-embedded exposure to health topics, rendering them particularly vulnerable to health misinformation and leaving critical knowledge gaps unaddressed [11]. Addressing these knowledge and behavioral gaps among young women aligns with Saudi Arabia's Vision 2030 healthcare priorities, which emphasize preventive healthcare, health promotion, and early detection strategies to improve population health outcomes. Previous studies in Saudi Arabia and the broader region have shown that non-medical female university students demonstrate limited knowledge of breast cancer risk factors, symptoms, and early detection approaches, which may contribute to delayed recognition and healthcare-seeking behaviors [12-15]. This population is thus identified as a higher-risk group for low health literacy regarding breast cancer, necessitating targeted assessment and intervention [16].

The present study addresses this gap by providing a comprehensive, multi-domain assessment of breast cancer awareness, knowledge of risk factors and warning signs, and self-reported breast health practices, including awareness of screening and clinical evaluation approaches, among non-medical female university students in Riyadh, the most populous city in Saudi Arabia and the hub of its higher education sector. A particular focus is placed on understanding factors associated with breast cancer-related knowledge, awareness, and self-reported health practices, and on identifying sociodemographic predictors associated with differences in these domains. The findings may contribute to the development of targeted, university-based educational strategies aimed at improving breast cancer-related awareness, knowledge, and health practices among non-medical female university students.

## Materials and methods

Study design and setting

This cross-sectional analytical study was conducted to assess breast cancer awareness, knowledge of risk factors and signs, attitudes toward early detection approaches, and self-reported breast health practices among non-medical female university students in Riyadh, Saudi Arabia. Riyadh, the capital and most populous city of KSA, had an estimated population of 8.6 million as of 2019 and is home to numerous public and private higher education institutions [17].

Eligibility criteria

Inclusion criteria: Non-medical female students enrolled at public or private universities in Riyadh, irrespective of nationality.

Exclusion criteria: All male students; high school students; students enrolled in medicine, nursing, pharmacy, or any other healthcare-related degree; non-medical female students from outside Riyadh; and any individual who, despite meeting the inclusion criteria, declined written informed consent or failed to complete the questionnaire.

Sample size and sampling technique

The required sample size was estimated using the Raosoft online calculator, assuming a 5% margin of error and a 95% confidence level, yielding a minimum of 525 participants. Applying a design effect of 1.3 to account for potential intraclass correlation resulting from the multistage university-based sampling structure increased the adjusted minimum sample size requirement to 683 participants. A final sample of 679 students was enrolled, providing adequate precision for subgroup analyses. To minimize potential response bias, questionnaires were completed anonymously, confidentiality was assured, and standardized instructions were provided to encourage independent responses.

Sampling proceeded in two stages. First, Riyadh universities were stratified by sector into public (n = 5) and private (n = 11) [18,19], from which four universities were randomly selected: two private universities, Alfaisal University (AU) and AlMaarefa University (MU), and two public universities, King Saud University (KSU) and Princess Nourah bint Abdulrahman University (PNU). Second, within each university, participants were selected by proportional convenience sampling based on availability and willingness until the institutional quota was reached. Institutional quotas were determined proportionally according to the estimated distribution of eligible non-medical female students within each selected university.

Data collection instrument

A structured, self-administered questionnaire was adapted from previously published regional and international instruments [2,4,10,20-23]. Following cultural and linguistic adaptation, the instrument was translated from English into Arabic and back-translated into English using a standardized forward-backward process to ensure semantic equivalence. It was piloted among approximately 5% of the intended sample (n = 35) and reviewed by three content experts for clarity, relevance, readability, and content appropriateness prior to full administration. The 35 participants involved in pilot testing were excluded from the final analytical sample and were not included in the reported results.

The final version of the questionnaire consisted of 58 questions and 6 sections, which are: Sociodemographic characteristics (7 questions), general awareness of the prevalence and severity of breast cancer (10 questions), knowledge of risk factors and symptoms (17 questions), awareness of breast cancer early detection approaches and BSE (nine questions), attitudes toward breast cancer screening (eight questions), and sources of breast cancer information (seven questions). The complete questionnaire, domain composition, correct-answer key, scoring framework, and classification criteria are provided in the Appendix to facilitate reproducibility. The supplementary file includes the allocation of individual items to each domain, the expected correct responses for knowledge-related items, and the scoring approach used for each section. Binary scoring (1 = correct; 0 = incorrect or unsure) was applied to knowledge-related items. Negatively phrased items were reverse-coded before calculating composite scores. The resulting knowledge score ranged from 0 to 43, with higher scores indicating greater breast cancer-related knowledge. The eight-item attitude section used a five-point Likert scale (1 = strongly disagree to 5 = strongly agree), with scores standardized to a 0-100 scale. A threshold of 60% was applied as the cut-off for classifying responses as "sufficient" (>60%) or "insufficient" (≤60%), consistent with thresholds commonly used in comparable regional knowledge-attitude-practice studies. This approach was selected to provide a standardized classification of participants achieving a majority of correct responses. Data collection was conducted between 6 July and 25 September 2025 by a team of trained medical students from participating universities. The questionnaire was administered through Google Forms during supervised in-person data collection sessions. All questions were designated as required fields to minimize incomplete responses.

The sociodemographic section included questions such as age, nationality, university name, educational program, academic year, family history of breast cancer, and individual monthly income. The awareness sections, including a total of 43 yes/no questions, focused on participants' general knowledge and understanding of breast cancer. These sections assessed participants' awareness of the disease and topics such as the global and national prevalence of breast cancer, its severity, recognition of common symptoms, the importance of early detection and appropriate screening recommendations, and awareness of major risk factors, such as family history, obesity, hormonal exposure, and early puberty. In addition, the questionnaire explored whether participants attended awareness campaigns or received any form of education about breast cancer, as well as their personal engagement in screening practices and their preferences regarding the gender of healthcare providers performing the examinations. Some questions assessed participants' awareness and perceptions regarding different breast health approaches, including mammography, CBE, and BSE. The attitude section consisted of eight questions assessing the participants' ability to recognize warning signs and perform BSE, their beliefs regarding the accessibility and importance of mammography and CBE, and the importance of regular annual screening after the age of 40. Additionally, this section included items related to emotional and psychological barriers to examination, such as embarrassment and fear of diagnosis.

Data analysis

Data were entered and analyzed using the Statistical Product and Service Solutions (SPSS, version 27.0; IBM SPSS Statistics for Windows, Armonk, NY). No missing data were present in the final dataset because all questionnaire items were configured as mandatory fields in Google Forms (Alphabet Inc., Mountain View, CA), preventing submission of incomplete responses. Data collection was conducted in person, allowing the research team to monitor questionnaire completion. Categorical variables were summarized as frequencies and percentages. Continuous variables were tested for normality using the Shapiro-Wilk test and Quantile-Quantile (Q-Q) plot inspection; given non-normal distributions, data were expressed as the median with interquartile range (IQR). Group comparisons of knowledge scores were performed using the Mann-Whitney U test (two groups) and the Kruskal-Wallis test (three or more groups), with Bonferroni-corrected post-hoc pairwise comparisons where the omnibus test was significant. Chi-square tests examined associations between categorical sociodemographic variables and dichotomized outcome domains (sufficient/insufficient awareness, knowledge, and screening practice). Binary logistic regression analyses were performed to identify independent predictors of each outcome domain. Variables included in the multivariable regression models were selected based on clinical relevance and those demonstrating significant associations in preliminary analyses. The models were not adjusted for clustering at the university level; therefore, this limitation was considered when interpreting the findings. Given the exploratory nature of the regression analyses, the reported odds ratios should be interpreted as associations rather than causal effects. Results were reported as odds ratios (ORs) with 95% confidence intervals (CIs). Statistical significance was set at p < 0.05 throughout.

Ethical considerations

This study was conducted in accordance with the Declaration of Helsinki. Ethical approval was granted by the Institutional Review Board (IRB) of AlMaarefa University (Ref. No: IRB25-068; date: 24 June 2025). All participants provided verbal and written informed consent before completing the questionnaire, were informed of their right to withdraw at any time without consequence, and were assured of full data anonymization and secure storage accessible only to the core research team.

## Results

Sociodemographic characteristics

As depicted in Table 1, out of the total number of participants (n = 679), most of them were aged 20-22 years (46.1%, n = 313), Saudi nationals (91.2%, n = 619), from PNU (33.1%, n = 224), enrolled bachelor's programs (91.3%, n = 620), in their second years (28.9%, n = 196), with less than 1,000 Saudi Riyals monthly income (57.4%, n = 390), and over two-third (66.1%, n = 449) did not have a family member diagnosed with breast cancer.

**Table 1:** Sociodemographic characteristics of the participants. AU = Alfaisal University; MU = AlMaarefa University; KSU = King Saud University; PNU = Princess Nourah bint Abdulrahman University; SR = Saudi Riyals; BC = Breast Cancer

Variable	Category	No.	%
Age	19 or younger	227	33.4
	20-22 years	313	46.1
	23-25 years	103	15.2
	26 or older	36	5.3
Nationality	Saudi	619	91.2
	Non-Saudi	60	8.8
University name	AU	102	15.0
	MU	132	19.4
	KSU	221	32.5
	PNU	224	33.1
Educational program	Diploma	51	7.5
	Bachelor	620	91.3
	Master/PhD	8	1.2
Academic year	Preparatory year	30	4.4
	1^st^ year	120	17.7
	2^nd^ year	196	28.9
	3^rd^ year	170	25.0
	4^th^ year	152	22.4
	Postgraduate (master/PhD)	11	1.6
Individual monthly income	Less than 1,000 SR	390	57.4
	From 1,000 to 5,000 SR	231	34.0
	From 5,000 to 10,000 SR	31	4.6
	More than 10,000 SR	27	4.0
Family history of BC	Yes	230	33.9
	No	449	66.1

Breast cancer knowledge about risk factors, signs, and screening practices

Risk Factors

The most frequently identified risk factors were family history (65.5%, n = 445) and increasing age (56.7%, n = 385), while recognition of less commonly recognized reproductive factors was limited: early puberty was correctly identified by only 16.2% (n = 110) of participants, nulliparity by 23.6% (n = 160), and menopausal-related risk factors by 27.2% (n = 185). Awareness of lifestyle-related risk factors was moderately low; only 50.8% (n = 345) correctly identified breastfeeding as a protective factor, suggesting limited recognition of protective reproductive factors.

Signs

Most participants correctly identified breast mass or swelling as signs (90.9%, n = 617), yet misconceptions were evident, with only 5.9% (n = 40) correctly identifying that pain is not a defining characteristic of an early breast cancer mass, indicating a potential misconception regarding symptom presentation. Similarly, only 13.8% correctly identified that chest pain during deep breathing is not a characteristic sign, reflecting substantial symptom misinformation.

Screening Methods and Practices

While 66.9% (n = 454) were aware that mammography is recommended annually for women over 40, only 51.0% (n = 347) recognized it as the most effective early detection tool, and only 35.2% (n = 239) knew where to access mammography services in Riyadh. Regarding BSE, 56.1% (n = 381) incorrectly believed that BSE alone is sufficient for breast cancer detection (reverse-scored), suggesting a misconception regarding the role of self-examination within breast cancer early detection approaches. Despite this, 40.4% (n = 274) reported having performed BSE, and 23.4% (n = 159) reported having undergone CBE. These findings indicate a discrepancy between breast cancer-related knowledge and self-reported breast health practices in this population.

Table 2 presents the frequency of correct responses across all knowledge domains.

**Table 2:** Frequency of correct answers regarding breast cancer risk factors, signs, and screening methods. ^A^ = Statement with reverse score

Variable	Category	No.	%
Risk factors	Increasing age of a woman	385	56.7
	Presence of a family history of breast cancer	445	65.5
	Lack of physical exercise	318	46.8
	Obesity	312	45.9
	Use of Contraceptive drugs	331	48.7
	Early puberty (below 12 years)	110	16.2
	Early menopause (before age 40)^ A^	185	27.2
	Young age of birth of the first child ^A^	273	40.2
	Giving birth to many children ^A^	307	45.2
	Never been pregnant	160	23.6
	Breastfeeding ^A^	345	50.8
	Radiation therapy to the chest	401	59.1
Signs	Mass or swelling in the breast	617	90.9
	Painful mass in the breast ^A^	40	5.9
	Bleeding or discharge from your nipple	493	72.6
	A nipple that suddenly turns inward (inverts)	380	56.0
	Chest pain while breathing deeply ^A^	94	13.8
Screening methods and practices	Breast self-exams alone are enough for breast cancer detection. ^A^	381	56.1
	Performed a breast self-examination.	274	40.4
	Mammography is the most effective tool for early detection of breast cancer.	347	51.0
	Mammograms are recommended annually for women over 40.	454	66.9
	Do you know where to access mammogram services in Riyadh?	239	35.2
	Performed a clinical breast exam to check for abnormalities.	159	23.4

Levels of awareness, knowledge, attitude, and screening practice of breast cancer

As illustrated in Figure 1, a difference was observed across the four outcome domains. While 85% (n = 578) of participants demonstrated sufficient attitude toward breast cancer screening, indicating broadly positive intentions, only 44% (n = 299) had adequate general awareness of the disease's prevalence and severity. The proportion decreased to 19% (n = 132) for sufficient knowledge of risk factors and signs and 23% (n = 158) for sufficient self-reported screening practice. This cascading gap, from high attitude to low practice, characterizes the knowledge-attitude-practice disconnect central to this study's findings.

**Figure 1:**
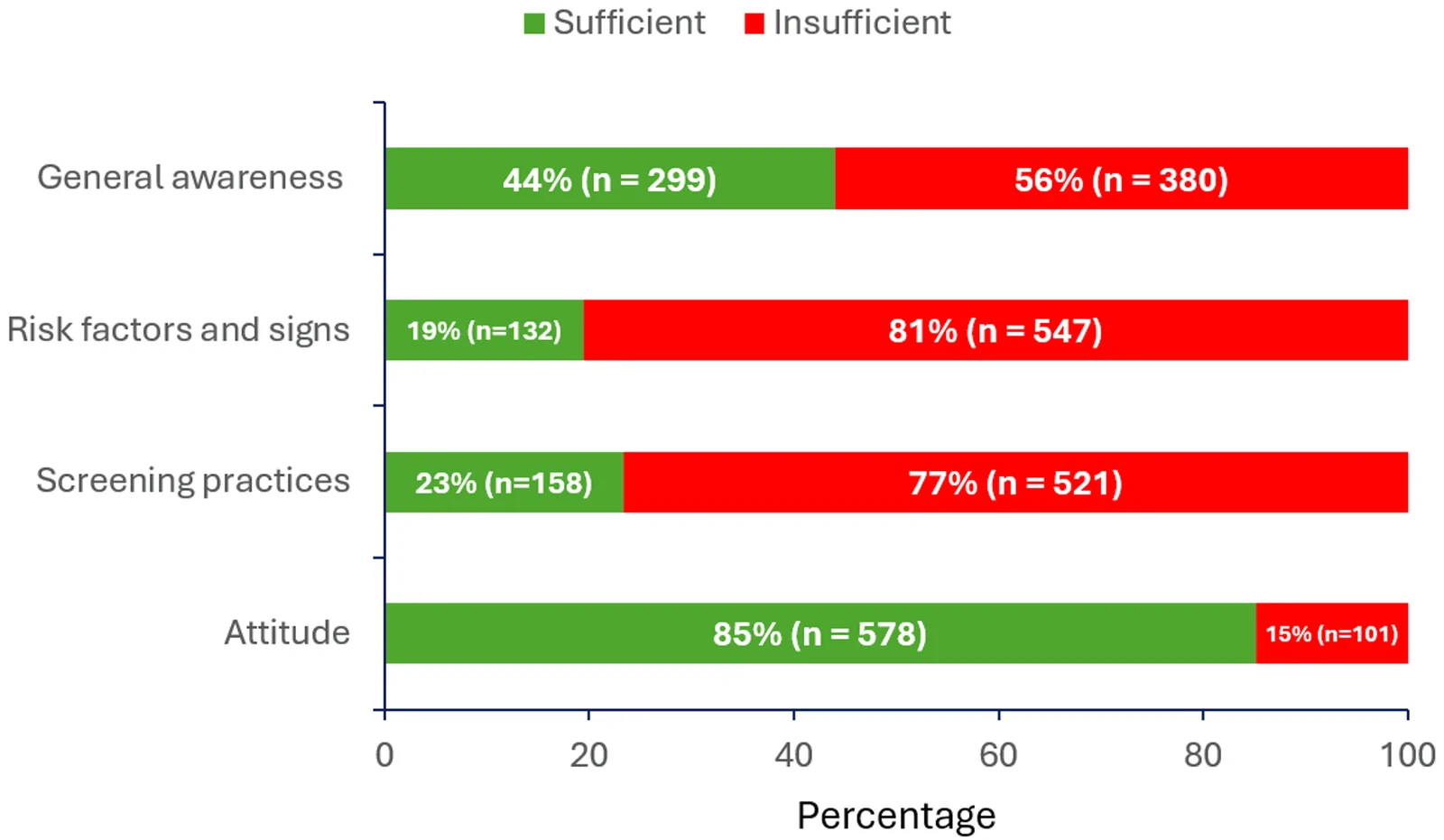
Levels of breast cancer awareness, knowledge, attitudes, and screening practices.

Overall knowledge scores by sociodemographic variables

The median total knowledge score was 19 (IQR: 16-22) out of a maximum of 43. Age was significantly associated with knowledge (p < 0.001): post-hoc analysis showed that participants aged 23-25 and ≥26 scored significantly higher than younger cohorts (adjusted p < 0.001), with no significant difference between the two older groups. University affiliation was significantly associated with knowledge scores (p < 0.001), with MU students achieving higher median scores (23 (IQR: 20-24.75)) compared to all other institutions. Educational program was also significant (p = 0.031), with bachelor's students outperforming diploma students (adjusted p = 0.046). Academic year was significant (p = 0.016), with first-year students scoring lower than third- and fourth-year students. Monthly income was associated with knowledge (p = 0.025), with the 5,000-10,000 SR bracket achieving the highest median scores. Both nationality (p = 0.028) and family history of breast cancer (p < 0.001) were significantly associated with higher knowledge, with non-Saudi participants and those reporting a positive family history scoring higher. Table 3 presents a comparison of total knowledge scores across sociodemographic variables.

**Table 3:** Comparing the total knowledge score across different sociodemographic variables. Categorical variables were presented as numbers and percentages. Continuous variables were presented as the median with the interquartile range. Statistical significance was set at p < 0.05. ^A^ = Kruskal-Wallis Test; ^B^ = Mann-Whitney Test

Variable	Category	Median total score	P value
Age	19 or younger	18 (16-21)	<0.001 ^A^
	20-22 years	18 (15-21)	
	23-25 years	20 (18-23)	
	26 or older years	22 (19-24)	
Nationality	Saudi	19 (16-21)	0.028 ^B^
	Non-Saudi	20 (18-23)	
University name	AU	18 (15.75-20)	<0.001 ^A^
	MU	23 (20-24.75)	
	KSU	18 (15-20)	
	PNU	18 (15-21)	
Educational program	Diploma	18 (15-19)	0.031 ^A^
	Bachelor	19 (16-22)	
	Master/PhD	20 (18.25-22.75)	
Academic year	Preparatory year	17.5 (15-20.25)	0.016 ^A^
	1^st^ year	17.5 (15-20)	
	2^nd^ year	19 (15-22)	
	3^rd^ year	19 (16-22)	
	4^th^ year	19 (17-22)	
	Postgraduate (master/PhD)	20 (15-20)	
Monthly income	Less than 1,000 SR	19 (16-21)	0.025 ^A^
	From 1,000 to 5,000 SR	19 (15-21)	
	From 5,000 to 10,000 SR	21 (18-23)	
	More than 10,000 SR	18 (15-20)	
Family history of BC	Yes	20 (17-23)	<0.001 ^B^
	No	18 (15-21)	

Association of different sociodemographic characteristics and a sufficient level of awareness, knowledge, and screening practice

Table 4 summarizes bivariate associations between sociodemographic characteristics and the three sufficient dichotomized outcome domains. Age was significantly associated with all three domains: general awareness (χ² = 8.853, df = 3, p = 0.031), knowledge (χ² = 11.533, df = 3, p = 0.009), and screening practice (χ² = 48.559, df = 3, p < 0.001), with higher sufficiency rates observed among older participants. University affiliation demonstrated significant associations across all three domains (p = 0.002 (χ² = 15.321, df = 3); p < 0.001 (χ² = 28, df = 3); p < 0.001 (χ² = 128.705)), with the highest sufficiency proportions observed among MU students. Family history of breast cancer was significantly associated with awareness (χ² = 8.376, df = 1, p = 0.004), knowledge (χ² = 8.570, df= 1, p = 0.003), with the largest difference observed for self-reported screening practice (χ² = 76.170, df = 1, p < 0.001), where 43.0% (n = 99) of students with a positive family history had sufficient practice versus only 13.1% (n = 59) without. Educational program was significantly associated with knowledge (χ² = 10.538, df = 2, p = 0.005) and screening practice (χ² = 8.583, df = 2, p = 0.014). The academic year was significantly associated with general awareness (χ² = 12.650, df = 5, p = 0.027) and screening practice (χ² = 15.188, df = 5, p = 0.010). Nationality and monthly income did not show significant bivariate associations with any of the three outcome domains at the p < 0.05 level.

**Table 4:** Association between sociodemographic characteristics and sufficient awareness, knowledge regarding risk factors and signs, and screening practices of breast cancer. Statistical significance was set at p < 0.05.

Variables	Category	Sufficient general awareness of BC			Sufficient knowledge of risk factors and signs			Sufficient screening practice		
		No.	%	P value	No.	%	P value	No.	%	P value
Age	≤19 years	98	43.2	0.031	37	16.3	0.009	28	12.3	< 0.001
	20-22 years	125	39.9		55	17.6		69	22.0	
	23-25 years	55	53.4		27	26.2		45	43.7	
	≥26 years	21	58.3		13	36.1		16	44.4	
Nationality	Saudi	269	43.5	0.330	117	18.9	0.254	139	22.5	0.107
	Non-Saudi	30	50.0		15	25.0		19	31.7	
University name	AU	41	40.2	0.002	13	12.7	< 0.001	17	16.7	< 0.001
	MU	78	59.1		47	35.6		80	60.6	
	KSU	92	41.6		35	15.8		33	14.9	
	PNU	88	39.3		37	16.5		28	12.5	
Educational Program	Diploma	18	35.3	0.253	7	13.7	0.005	5	9.8	0.014
	Bachelor	276	44.5		120	19.4		149	24.0	
	Master/PhD	5	62.5		5	62.5		4	50.0	
Academic Year	Preparatory year	7	23.3	0.027	4	13.3	0.074	1	3.3	0.010
	1st year	42	35.0		14	11.7		19	15.8	
	2nd year	92	46.9		37	18.9		45	23.0	
	3rd year	80	47.1		36	21.2		49	28.8	
	4th year	71	46.7		37	24.3		40	26.3	
	Master/PhD	7	63.6		4	36.4		4	36.4	
Monthly income	< 1,000 SR	174	44.6	0.179	65	16.7	0.100	85	21.8	0.702
	1,000 to 5,000 SR	96	41.6		50	21.6		57	24.7	
	5,000 to 10,000 SR	19	61.3		9	29.0		9	29.0	
	> 10,000 SR	10	37		8	29.6		7	25.9	
Family history of BC	Yes	119	51.7	0.004	59	25.7	0.003	99	43.0	< 0.001
	No	180	40.1		73	16.3		59	13.1	

Regression model between sociodemographic characteristics and awareness, knowledge, and screening practice of breast cancer

For general awareness, enrollment in the preparatory year was identified as a significant independent negative predictor (OR = 0.144; 95% CI: 0.022-0.922; p = 0.041), suggesting that students at the earliest stage of university engagement have the lowest awareness levels even after adjusting for other variables.

For knowledge of risk factors and signs, MU affiliation was significantly associated with higher odds of it (OR = 2.718; 95% CI: 1.398-5.282; p = 0.003). Conversely, enrollment in diploma programs (OR = 0.035; 95% CI: 0.003-0.505; p = 0.013) and bachelor's programs (OR = 0.050; 95% CI: 0.005-0.558; p = 0.015), relative to master's/PhD as the reference category, were significant negative predictors. A lower monthly income (< 1,000 SR) was also independently associated with lower knowledge (OR = 0.339; 95% CI: 0.127-0.904; p = 0.031).

In the multivariable logistic regression model, MU affiliation was significantly associated with higher odds of sufficient self-reported screening practice (OR = 5.488; 95% CI: 2.863-10.519; p < 0.001). A positive family history of breast cancer was also significantly associated with higher odds of sufficient self-reported screening practice (OR = 2.384; 95% CI: 1.488-3.819; p < 0.001).

Table 5 presents the results of the multivariate binary logistic regression for each of the three outcome domains.

**Table 5:** Regression model between sociodemographic characteristics and awareness, knowledge, and screening practice of breast cancer. Statistical significance was set at p < 0.05. Ref = Reference; OR = Odds Ratio; CI = Confidence interval

Variables	Category	Awareness of BC		Knowledge of BC		Screening of BC	
		OR (95% CI)	P value	OR (95% CI)	P value	OR (95% CI)	P value
Age	≤19 years	1.325 (0.544-3.225)	0.536	1.551 (0.549-4.385)	0.408	0.714 (0.251-2.029)	0.527
	20-22 years	0.732 (0.328-1.633)	0.446	0.894 (0.367-2.179)	0.806	0.943 (0.386-2.305)	0.897
	23-25 years	0.935 (0.413-2.115)	0.872	0.815 (0.334-1.984)	0.652	1.253 (0.515-3.050)	0.619
	≥26 years	Ref	-	Ref	-	Ref	-
Nationality	Saudi	0.881 (0.497-1.559)	0.662	0.751 (0.381-1.480)	0.409	1.056 (0.523-2.135)	0.879
	Non-Saudi	Ref	-	Ref	-	Ref	-
University name	AU	1.126 (0.668-1.897)	0.657	0.516 (0.241-1.107)	0.089	1.340 (0.654-2.746)	0.424
	MU	1.748 (0.997-3.066)	0.051	2.718 (1.398-5.282)	0.003	5.488 (2.863-10.519)	< 0.001
	KSU	1.186 (0.798-1.764)	0.398	0.963 (0.570-1.629)	0.890	1.144 (0.651-2.010)	0.640
	PNU	Ref	-	Ref	-	Ref	-
Educational Program	Diploma	0.559 (0.076-4.135)	0.569	0.035 (0.003-0.505)	0.013	0.085 (0.007-1.014)	0.051
	Bachelor	0.725 (0.107-4.884)	0.741	0.050 (0.005-0.558)	0.015	0.194 (0.020-1.876)	0.157
	Master/PhD	Ref	-	Ref	-	Ref	-
Academic Year	Preparatory Year	0.144 (0.022-0.922)	0.041	1.416 (0.108-18.564)	0.791	0.237 (0.014-4.028)	0.319
	1st year	0.290 (0.052-1.631)	0.160	1.457 (0.122-17.436)	0.766	1.372 (0.152-12.375)	0.778
	2nd year	0.522 (0.097-2.798)	0.448	2.323 (0.209-25.820)	0.493	1.292 (0.153-10.896)	0.814
	3rd year	0.565 (0.105-3.031)	0.505	2.695 (0.242-29.997)	0.420	1.371 (0.162-11.636)	0.773
	4th year	0.572 (0.108-3.018)	0.510	3.735 (0.344-40.591)	0.279	1.175 (0.141-9.794)	0.881
	Master/PhD	Ref	-	Ref	-	Ref	-
Monthly income	< 1,000 SR	1.274 (0.532-3.054)	0.587	0.339 (0.127-0.904)	0.031	0.520 (0.188-1.440)	0.208
	1,000-5,000 SR	1.158 (0.480-2.797)	0.744	0.543 (0.204-1.445)	0.222	0.627 (0.226-1.741)	0.371
	5,000-10,000 SR	2.549 (0.835-7.780)	0.100	0.614 (0.176-2.148)	0.446	0.727 (0.195-2.715)	0.636
	> 10,000 SR	Ref	-	Ref	-	Ref	-
Family history of BC	Yes	1.320 (0.894-1.948)	0.162	1.152 (0.699-1.900)	0.579	2.384 (1.488-3.819)	< 0.001
	No	Ref	-	Ref	-	Ref	-

## Discussion

This study evaluated breast cancer awareness, knowledge, attitudes, and early detection-related practices among non-medical female university students in Riyadh, Saudi Arabia. The findings demonstrated a discrepancy between positive attitudes toward breast cancer early detection and lower levels of knowledge and reported practices. Although 85% (n = 578) of participants demonstrated sufficient attitudes, only 19% (n = 132) achieved sufficient knowledge, and 23% (n = 158) reported sufficient self-reported practice. This pattern highlights that positive attitudes alone may not translate into informed health behaviors without adequate understanding of breast cancer risk factors, symptoms, and appropriate healthcare pathways.

The observed knowledge-practice gap is consistent with findings among university student populations in different settings. In Saudi Arabia, Islam et al. reported differences in breast cancer knowledge between health and non-health college students, with non-health students demonstrating lower knowledge levels, highlighting the vulnerability of students without healthcare-related educational exposure [24]. Similar patterns have been observed internationally; Sarker et al. reported low breast cancer knowledge and limited breast self-examination practices among female university students in Bangladesh, with family history being associated with higher knowledge and practice scores [25]. These findings suggest that university students represent an important target population for interventions addressing specific informational and behavioral barriers rather than awareness alone.

Participants most frequently recognized family history (65.5%, n = 445) and increasing age (56.7%, n = 385) as breast cancer risk factors, consistent with previous literature identifying these as commonly recognized factors [23,26]. However, recognition of less commonly discussed reproductive factors was limited, including early puberty (16.2%, n = 110) and nulliparity (23.6%, n = 160). Similar gaps in recognizing breast cancer risk factors have been reported among female university students. Rahman et al. found that, although most University of Sharjah students had heard of breast cancer, knowledge regarding risk factors and warning signs remained incomplete, particularly among non-medical students [14]. This suggests that general awareness of breast cancer does not necessarily translate into accurate understanding of disease determinants.

Recognition of common breast cancer signs was relatively high, with breast mass or swelling identified by 90.9% (n = 617) of participants and nipple discharge by 72.6% (n = 493). However, only 5.9% (n = 40) correctly identified that a painful breast mass is not a typical early presentation, suggesting a misconception regarding symptom characteristics. Because this study assessed knowledge rather than healthcare-seeking behavior, the extent to which these misconceptions influence clinical evaluation cannot be determined. Reported engagement with breast health practices was lower than attitudes toward early detection. Although 40.4% (n = 274) reported performing BSE and 23.4% (n = 159) reported undergoing CBE, these findings should be interpreted cautiously. The majority of participants were young university students, and many were below the recommended age for routine mammographic screening. Furthermore, BSE, CBE, and mammography represent different components of breast health assessment and should not be considered equivalent screening modalities. Previous studies have similarly demonstrated differences between breast cancer awareness and practice. Alqahtani et al. reported high awareness of BSE but low regular practice in the Asir region [27], while Al-Zalabani et al. reported comparable differences between BSE and CBE practices in Madinah [28]. These findings emphasize the importance of improving understanding of appropriate breast health behaviors according to age and clinical recommendations. Comparable findings have been reported among female university students in Arab countries. Gamal et al. observed that although many students recognized breast cancer and BSE, regular BSE practice remained limited, indicating persistent gaps between awareness and behavioral application [29]. Similarly, Simegn et al. demonstrated that, among female university students, screening practices were associated with factors such as training, attitudes, and behavioral intentions, suggesting that knowledge alone may not be sufficient to influence practice [30].

University affiliation was associated with differences in breast cancer knowledge and self-reported practices. MU students demonstrated higher scores across several domains, and MU affiliation was associated with higher odds of sufficient knowledge (OR = 2.718; p = 0.003) and sufficient self-reported practice (OR = 5.488; p < 0.001). However, these findings should be interpreted cautiously because university-level factors, including educational exposure, health promotion activities, and healthcare access, were not directly measured. A positive family history of breast cancer was also associated with higher odds of sufficient self-reported practice (OR = 2.384; p < 0.001). This finding is consistent with studies among university students showing that family history may influence breast cancer-related awareness and preventive behaviors. Sarker et al. reported higher knowledge scores among students with a family history of breast cancer, while Osei et al. similarly identified family history as an important factor associated with breast cancer risk perception among female university students [25,31]. However, the mechanisms underlying this association cannot be determined from this cross-sectional study.

Strengths and limitations

This study offers several methodological strengths: a large, proportionally distributed multi-institutional sample; in-person, face-to-face data collection, minimizing missing data and response bias; use of a validated bilingual instrument developed through rigorous forward-backward translation; comprehensive domain coverage across awareness, knowledge, attitudes, and practice; and the application of appropriate non-parametric and multivariate analytical methods.

However, several limitations should be considered when interpreting these findings. First, the cross-sectional design prevents establishing causal relationships between sociodemographic characteristics, knowledge, attitudes, and reported practices. Second, the outcomes were based on self-reported questionnaire responses and may be affected by recall bias or social desirability bias. Although the final analyzed sample size was documented, participant flow information, including the exact number of students approached and declined participation, was not prospectively recorded. Therefore, the potential impact of non-response bias cannot be fully assessed. Additionally, classification of awareness, knowledge, attitudes, and practices relied on predefined scoring thresholds. Therefore, outcome misclassification is possible if participants misunderstood individual questionnaire items or if the selected thresholds did not fully capture clinically meaningful differences. Third, the age distribution of participants should be considered when interpreting screening-related findings. Most participants were younger than the recommended age for routine mammographic screening. Consequently, lower mammography-related practice rates may partly reflect age ineligibility rather than insufficient engagement with preventive healthcare. Fourth, although participants were recruited from multiple universities, convenience sampling was used within selected institutions. This may have introduced selection bias and limits the generalizability of findings beyond non-medical female university students in Riyadh. Fifth, participants were clustered within universities, and the regression analyses did not account for potential within-university correlation. Therefore, assoiations involving university affiliation should be interpreted cautiously. Finally, although multivariable analyses adjusted for measured sociodemographic factors, residual confounding from unmeasured variables cannot be excluded. Factors such as previous breast health education, healthcare access, health literacy, and exposure to awareness campaigns were not directly assessed and may have influenced observed associations.

Recommendations

The findings support the need for continued improvement of breast cancer-related health education among non-medical university students. Educational initiatives may focus on improving knowledge of breast cancer risk factors, symptom recognition, appropriate early detection approaches, and awareness of available healthcare services. University-based health education programs may represent a practical approach for reaching young women; however, the effectiveness of such interventions requires evaluation through longitudinal or controlled studies. Future research should investigate institutional and individual factors associated with breast cancer-related knowledge and practices, including previous educational exposure, healthcare access, perceived risk, and barriers to appropriate healthcare utilization.

## Conclusions

This study identified a knowledge-attitude-practice gap regarding breast cancer among non-medical female university students in Riyadh, Saudi Arabia. Although attitudes toward breast cancer screening and early detection were generally positive, gaps were observed in knowledge of risk factors, warning signs, and self-reported breast health practices. Misconceptions regarding certain breast cancer-related symptoms and limited awareness of available screening services were identified as areas requiring further attention. AlMaarefa University affiliation was associated with higher knowledge and self-reported screening practice, while family history of breast cancer was associated with sufficient screening practice. These findings highlight the importance of strengthening breast cancer-related health education among university students through culturally appropriate and evidence-based approaches. Future longitudinal and intervention studies are needed to evaluate whether educational strategies can improve breast cancer knowledge and health-related behaviors over time.

## Figures and Tables

**Figure 2:**
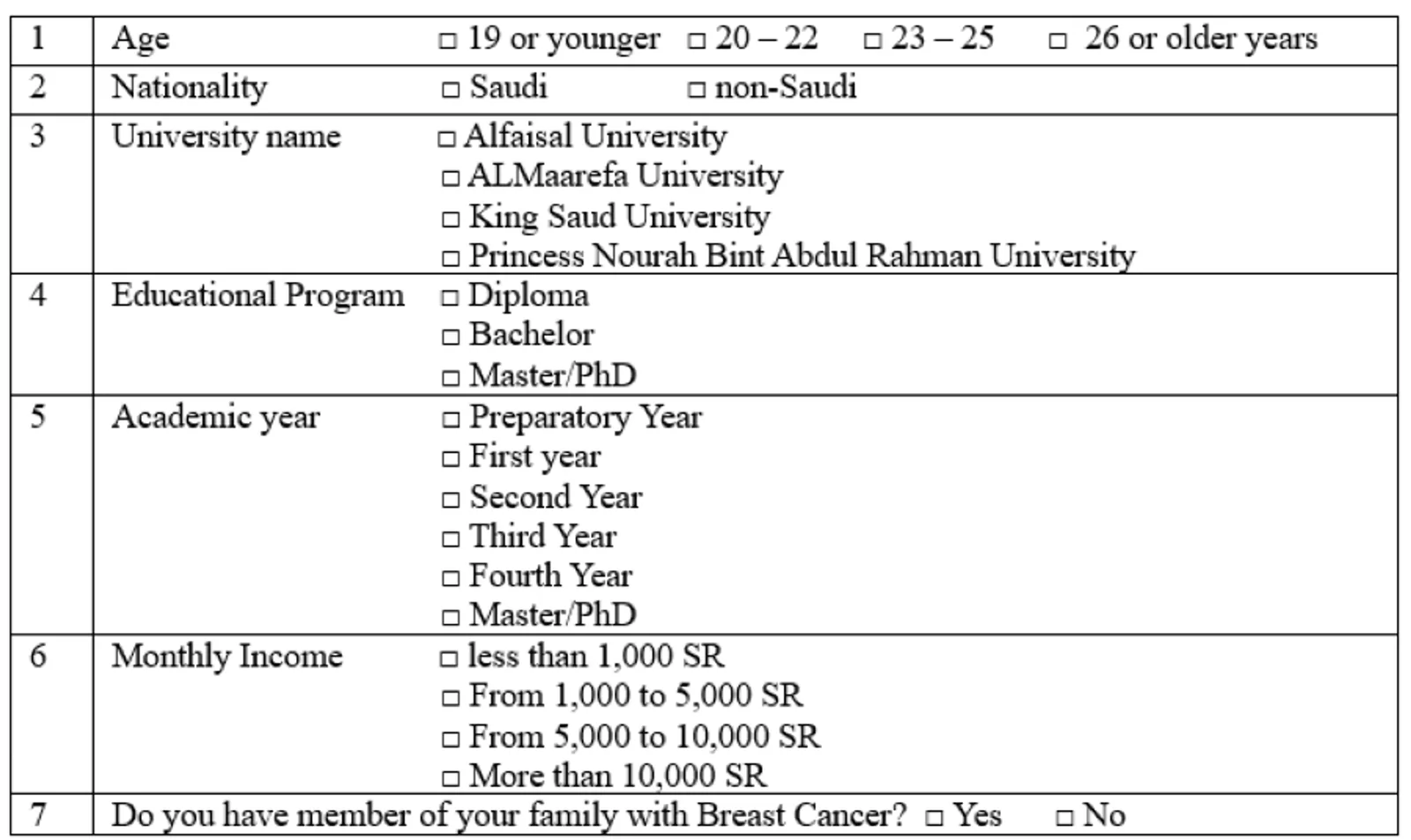
Section 1: Sociodemographic characteristics.

**Figure 3:**
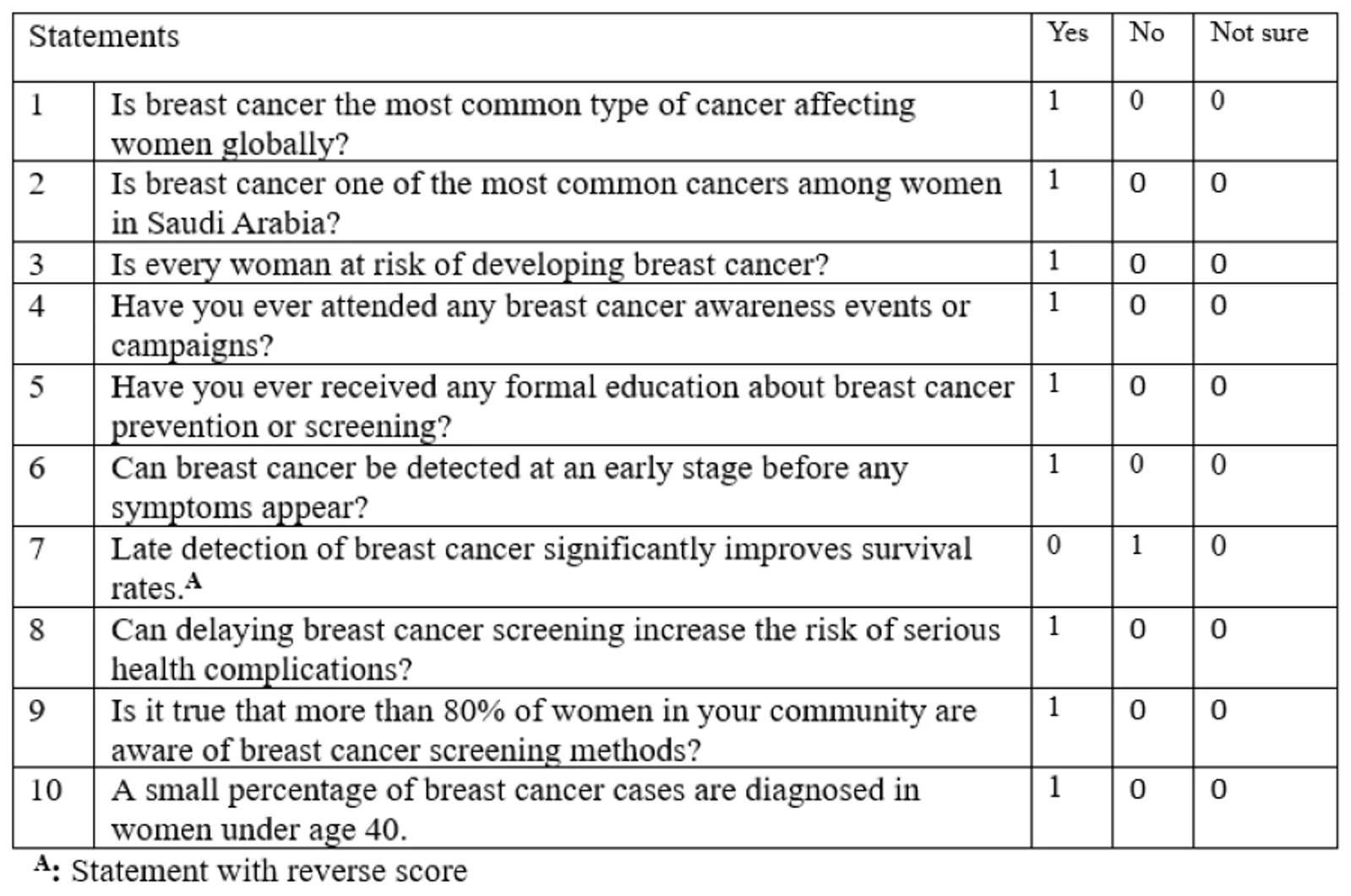
Section 2: General questions on breast cancer awareness. Please indicate whether the following statements are true by selecting "Yes," "No," or "Not sure."

**Figure 4:**
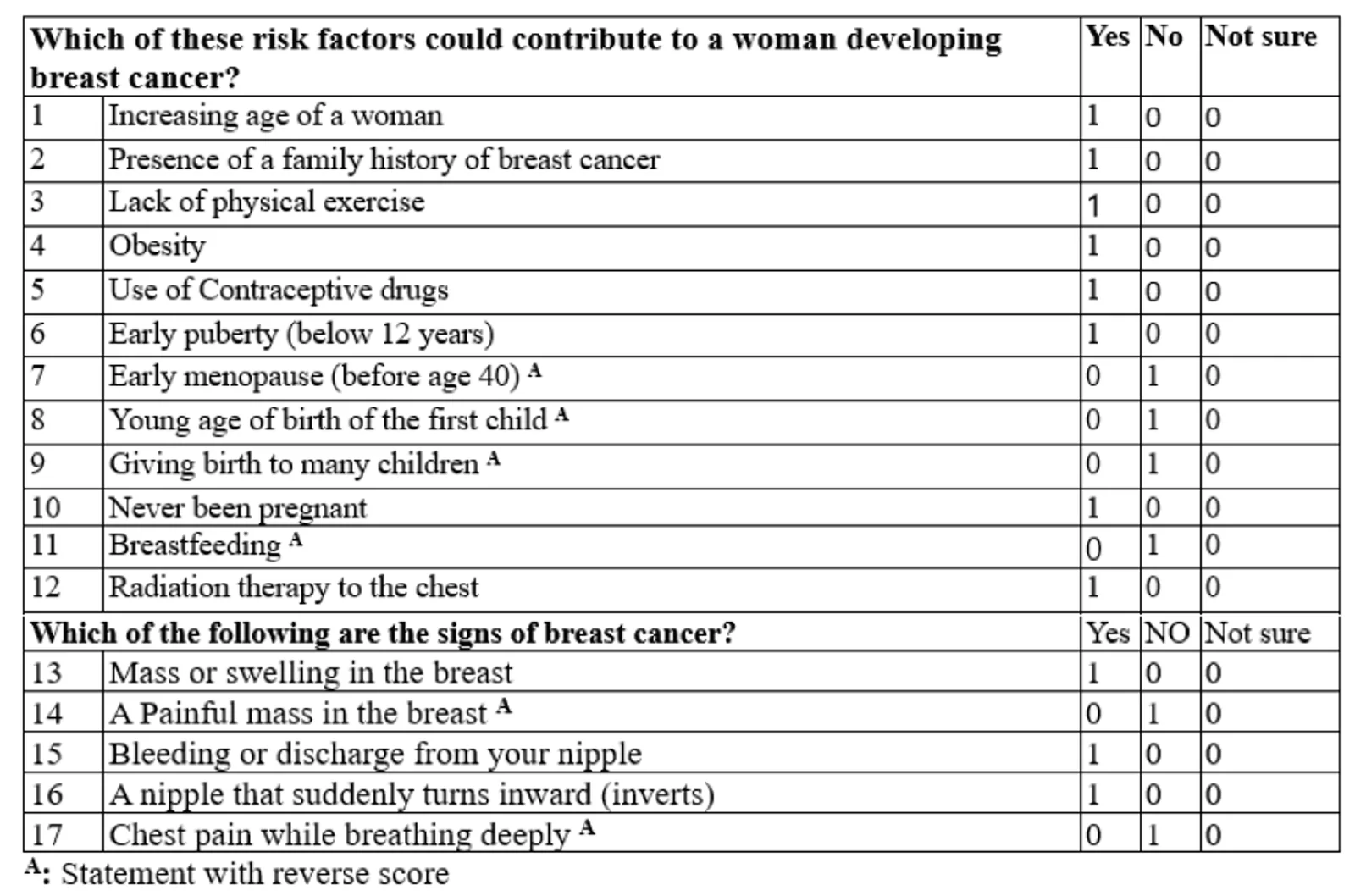
Section 3: Breast cancer risk factors and signs knowledge. Please indicate all risk factors you believe are related to breast cancer.

**Figure 5:**
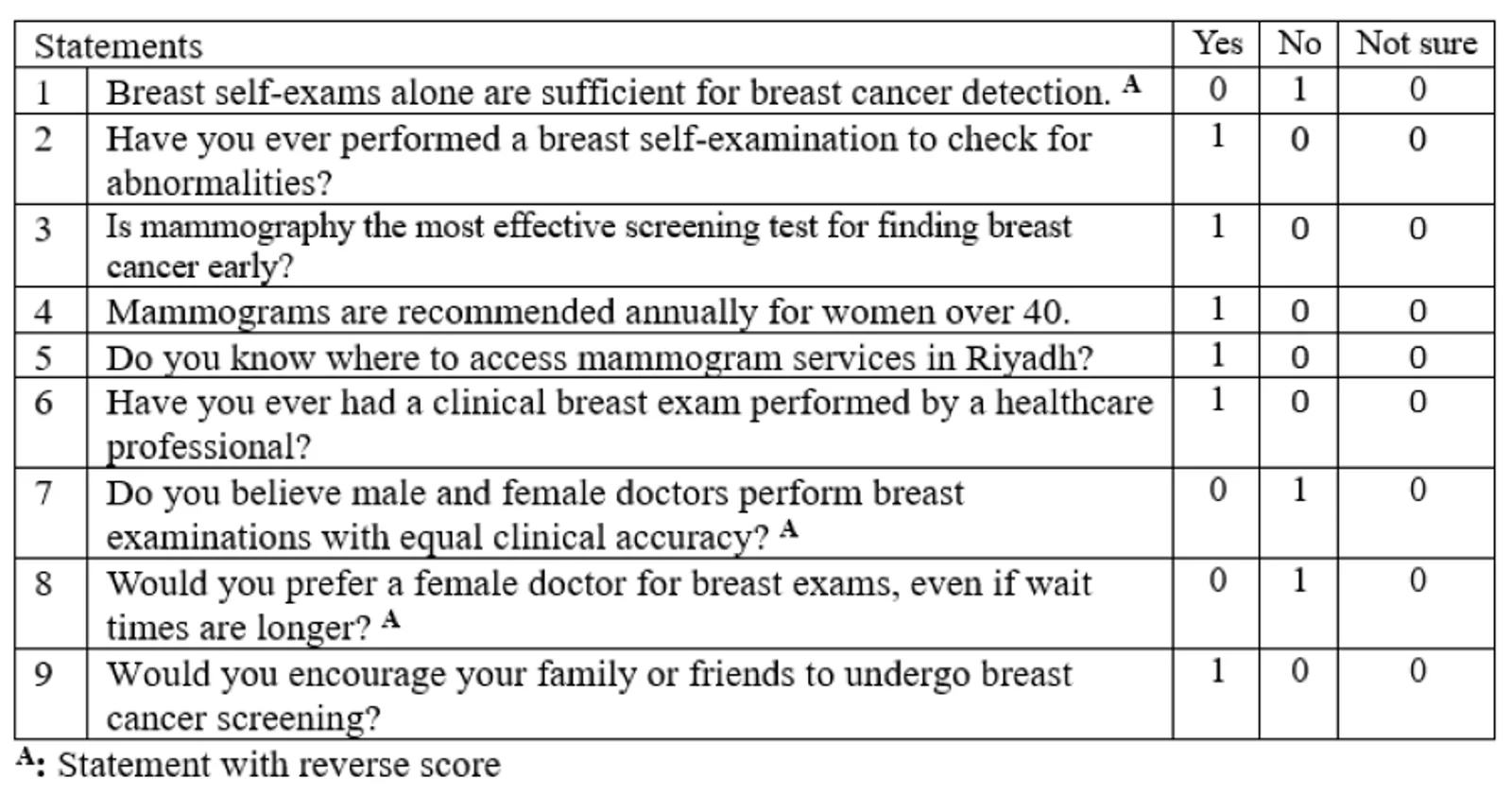
Section 4: Breast cancer screening method. Please indicate whether the following statements are true by selecting "Yes," "No," or "Not sure."

**Figure 6:**
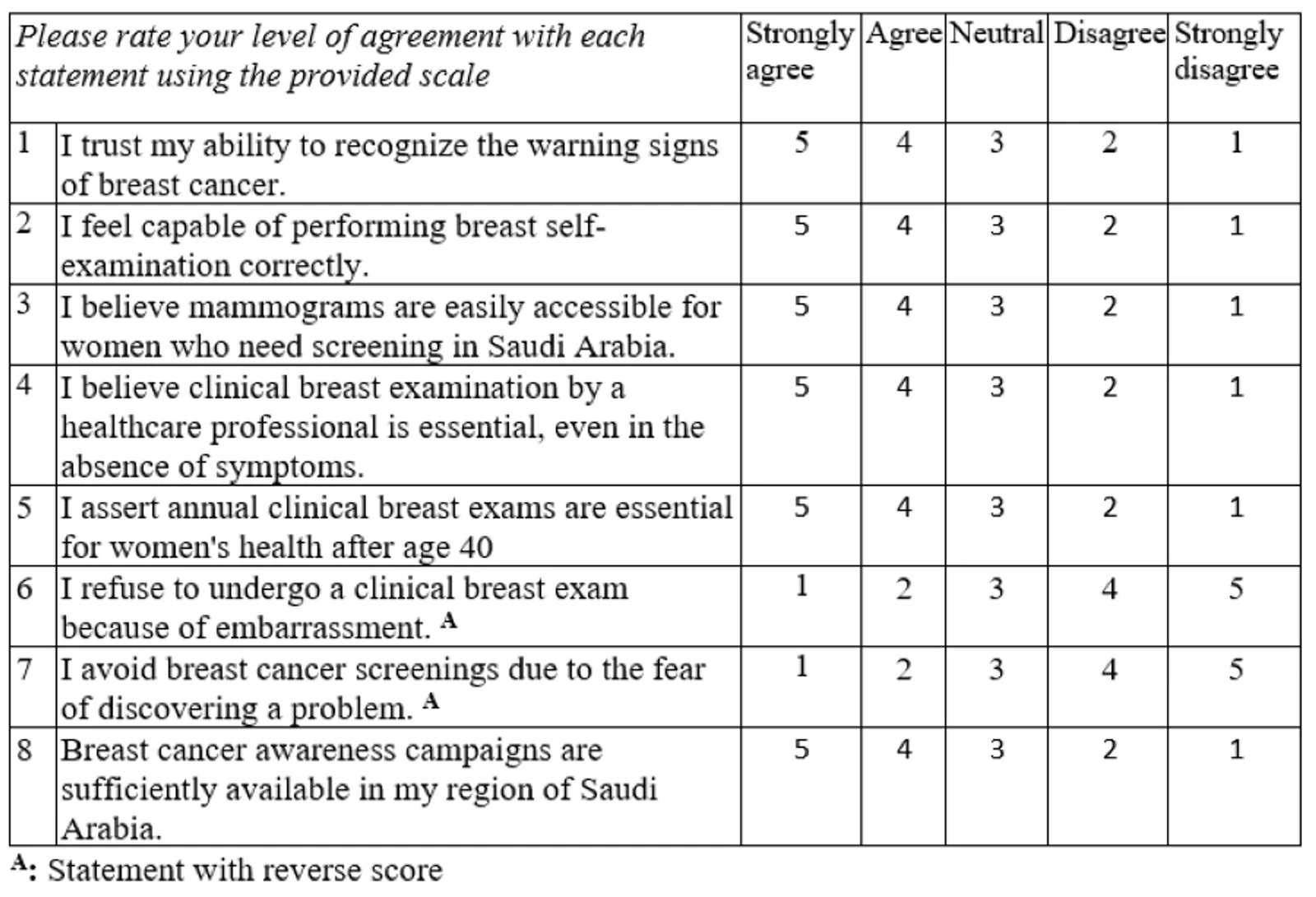
Section 5: Identify breast cancer attitudes.

**Figure 7:**
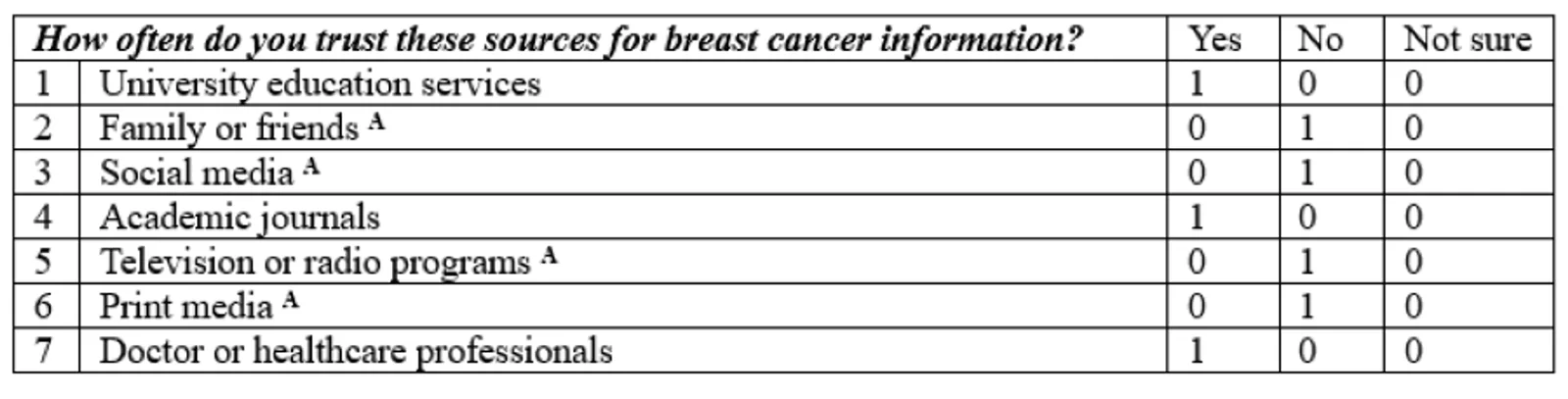
Section 6: Identify trusted sources of breast cancer information. Please indicate your primary sources of breast cancer information by using the provided scale. A: Statement with reverse score

**Figure 8:**
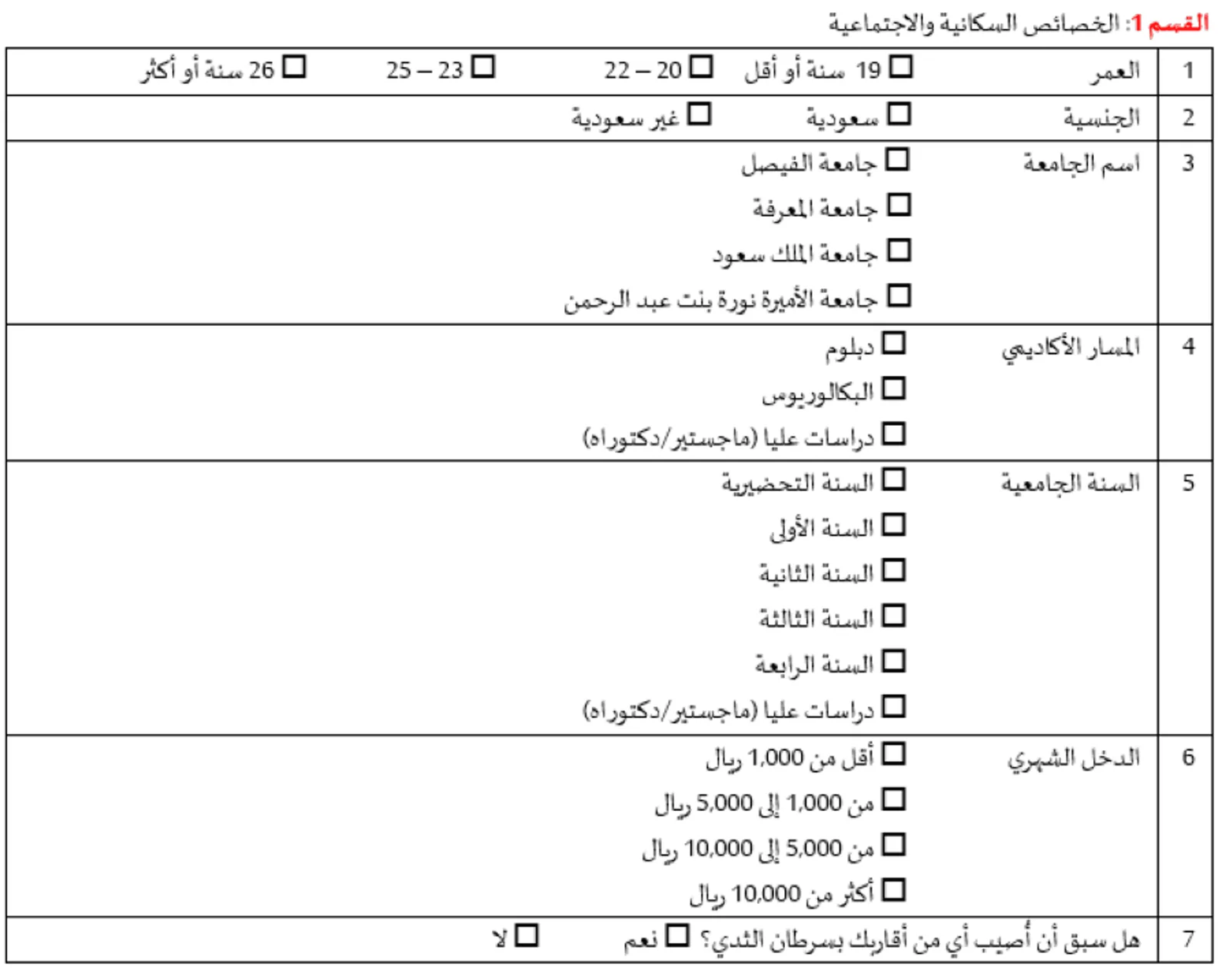
Section 1: Sociodemographic characteristics.

**Figure 9:**
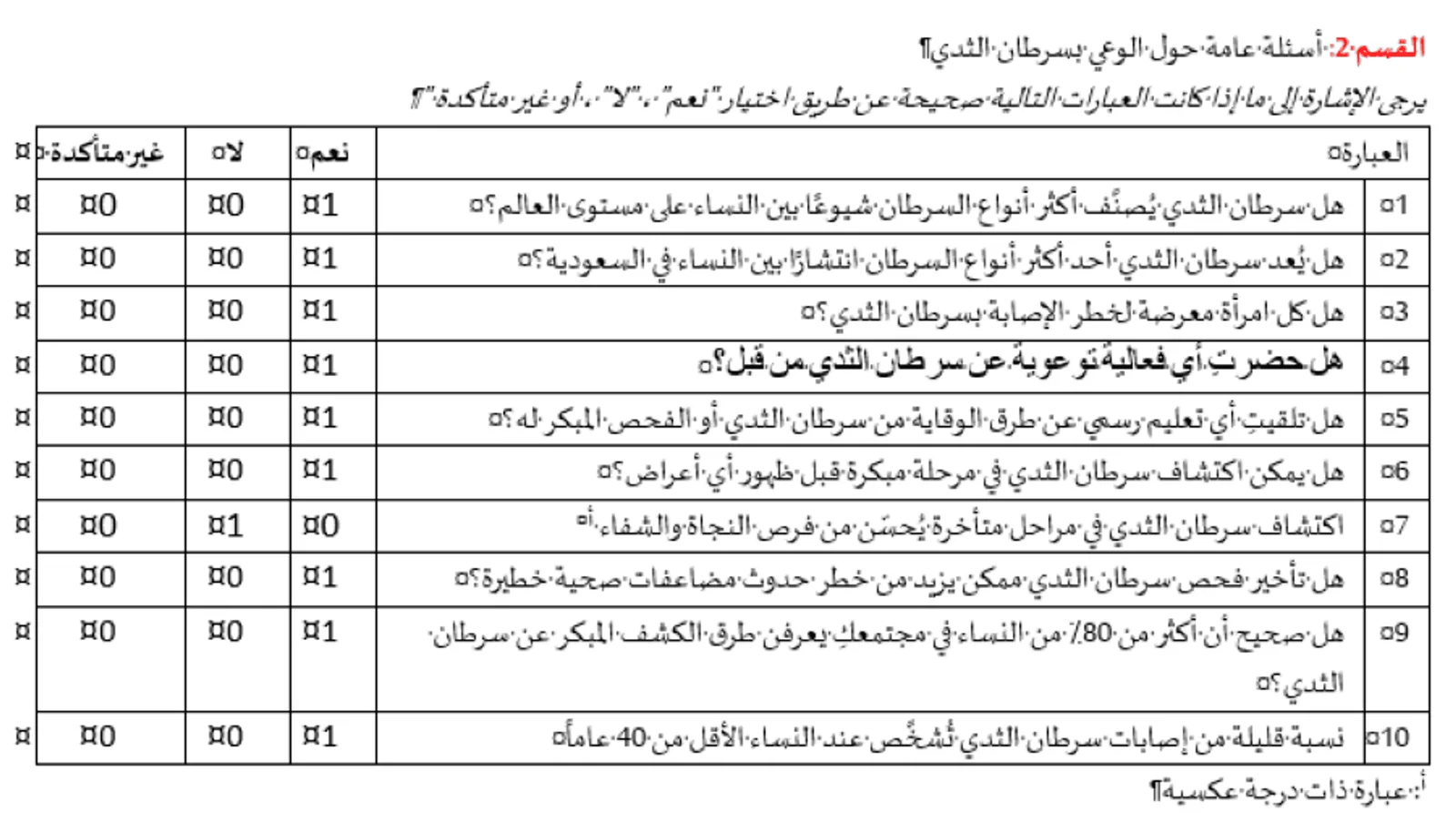
Section 2: General questions on breast cancer awareness.

**Figure 10:**
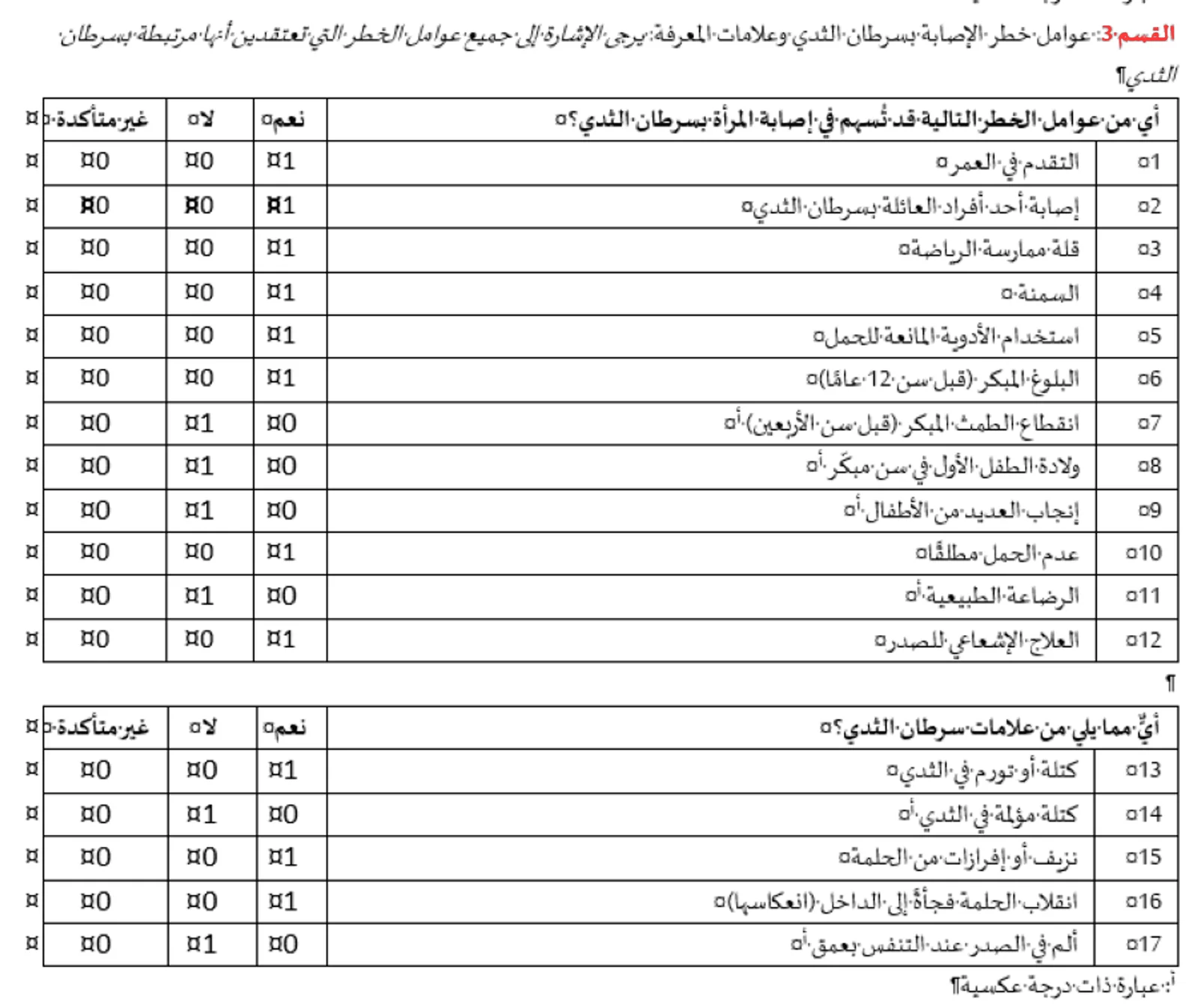
Section 3: Breast cancer risk factors and signs knowledge.

**Figure 11:**
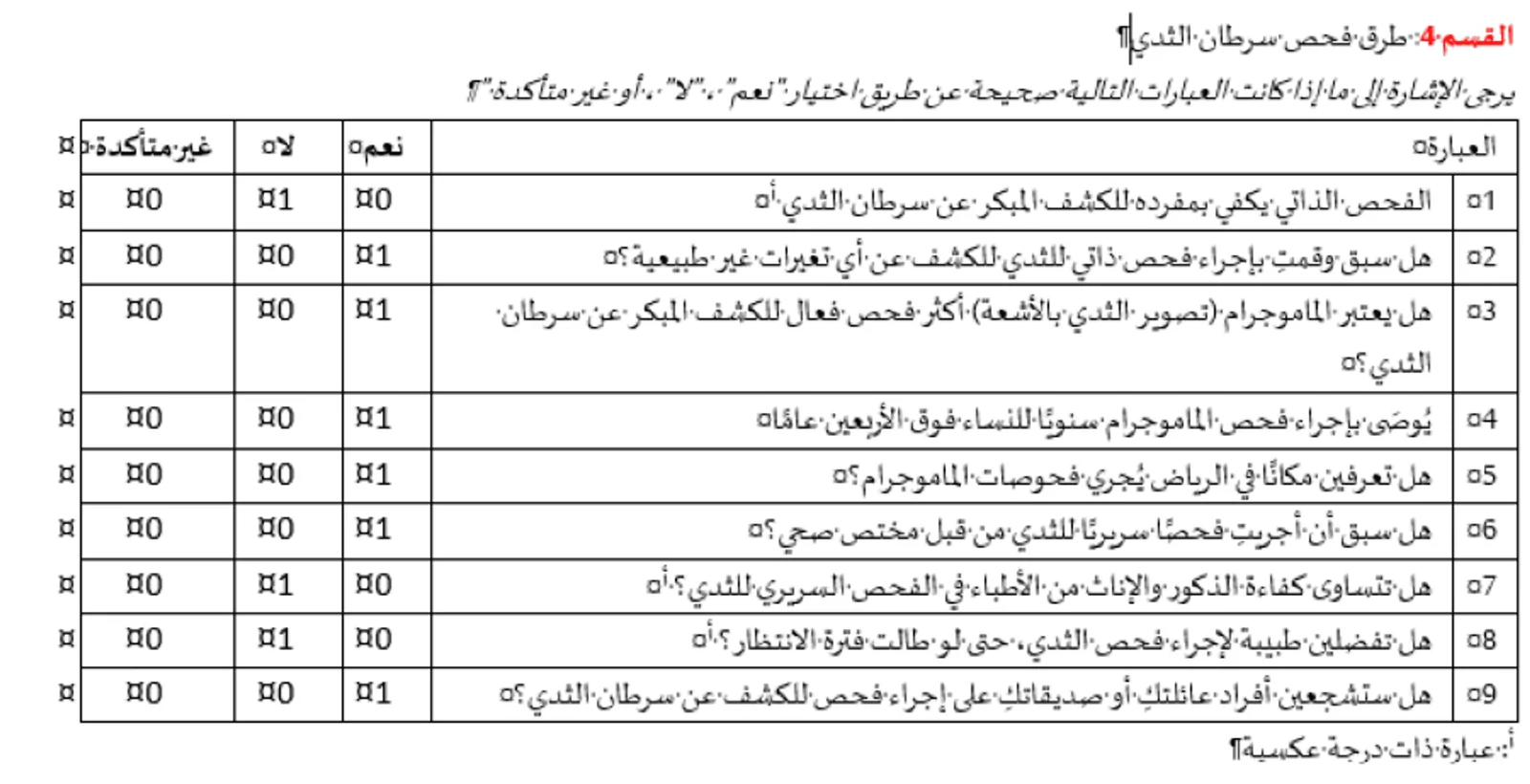
Section 4: Breast cancer screening method.

**Figure 12:**
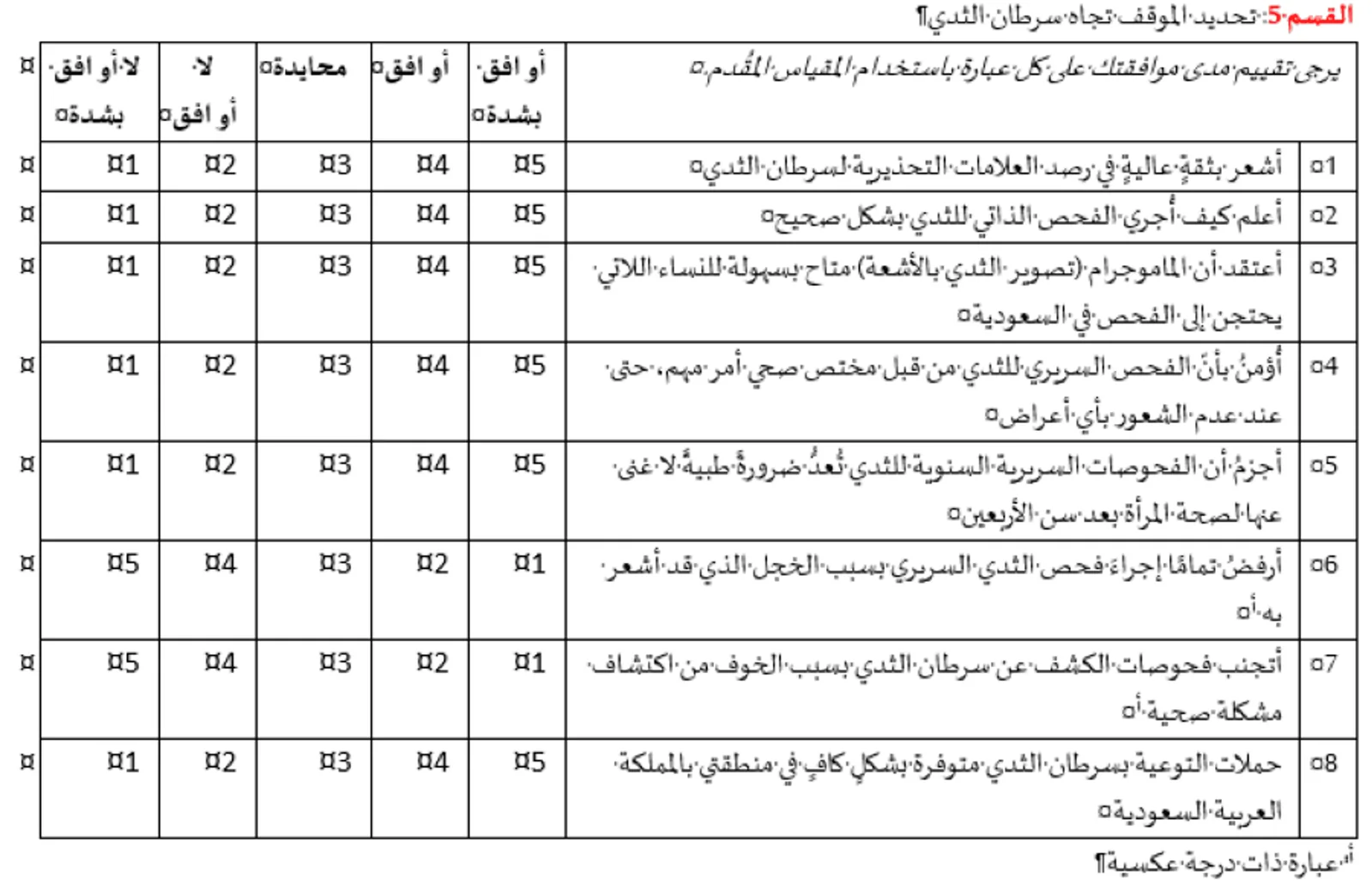
Section 5: Identify breast cancer attitudes.

**Figure 13:**
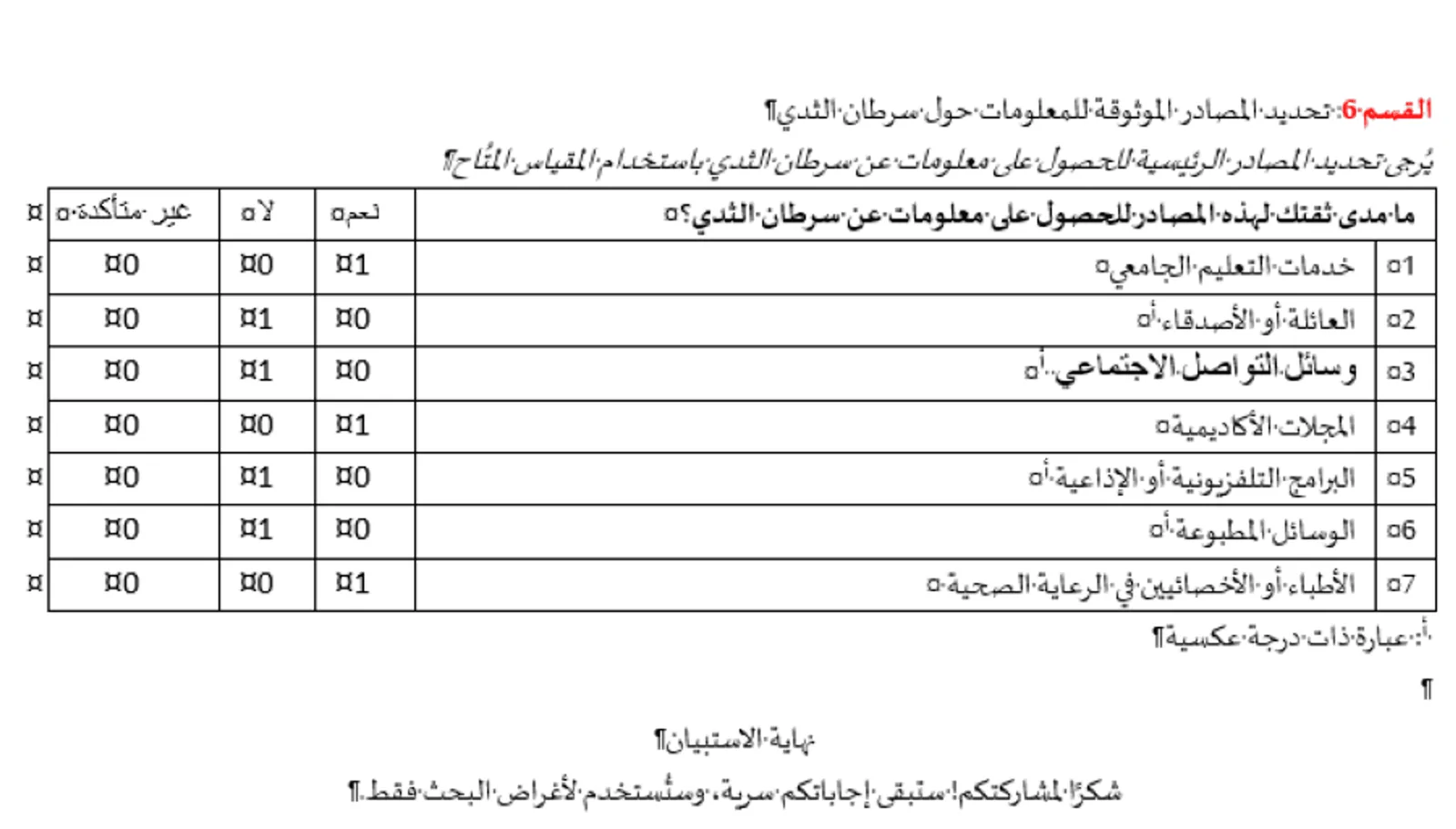
Section 6: Identify trusted sources of breast cancer information.

## Appendices

Appendix 1

English Questionnaire: "Bridging the Knowledge-Practice Gap in Breast Cancer Screening Among Non-medical Female University Students in Riyadh: A Cross-Sectional Study"

Dear Participant,

Thank you for participating in this survey. We are medical students from universities in Riyadh conducting a research study to assess breast cancer awareness, knowledge, attitudes, and early detection practices among female university students in Riyadh, Saudi Arabia. Your participation will help us better understand current levels of knowledge, perceptions, and behaviors related to breast cancer prevention and early detection. Please answer the questions honestly and as accurately as possible. All responses will remain confidential and will be used solely for research purposes.

Thank you for your participation! Your responses will remain confidential and will be used solely for research purposes.

Arabic Questionnaire

استبيان: "سد الفجوة بين المعرفة والممارسة في مجال الكشف المبكر عن سرطان الثدي لدى طالبات الجامعات غير المتخصصات في المجالات الطبية بمدينة الرياض: دراسة مقطعية"

عزيزتي المشاركة،

نشكركِ على تخصيص وقتكِ للمشاركة في هذا الاستبيان. نحن مجموعة من طلبة الطب من جامعات مدينة الرياض، ونجري دراسة بحثية تهدف إلى تقييم مستوى الوعي والمعرفة والاتجاهات والممارسات المتعلقة بالكشف المبكر عن سرطان الثدي لدى طالبات الجامعات في مدينة الرياض، المملكة العربية السعودية. ستساهم مشاركتكم في فهم مستوى المعرفة والتصورات والسلوكيات الحالية المتعلقة بالوقاية من سرطان الثدي والكشف المبكر عنه. يرجى الإجابة عن الأسئلة بكل صدق ودقة قدر الإمكان. ستظل جميع الإجابات سرية، وستُستخدم لأغراض البحث العلمي فقط.
